# Genome-wide identification of gene expression in contrasting maize inbred lines under field drought conditions reveals the significance of transcription factors in drought tolerance

**DOI:** 10.1371/journal.pone.0179477

**Published:** 2017-07-12

**Authors:** Xiaojing Zhang, Xuyang Liu, Dengfeng Zhang, Huaijun Tang, Baocheng Sun, Chunhui Li, Luyang Hao, Cheng Liu, Yongxiang Li, Yunsu Shi, Xiaoqing Xie, Yanchun Song, Tianyu Wang, Yu Li

**Affiliations:** 1 Institute of Crop Science, Chinese Academy of Agricultural Sciences, Beijing, China; 2 Institute of Grain Crops, Xinjiang Academy of Agricultural Sciences, Urumqi, China; Louisiana State University College of Agriculture, UNITED STATES

## Abstract

Drought is a major threat to maize growth and production. Understanding the molecular regulation network of drought tolerance in maize is of great importance. In this study, two maize inbred lines with contrasting drought tolerance were tested in the field under natural soil drought and well-watered conditions. In addition, the transcriptomes of their leaves was analyzed by RNA-Seq. In total, 555 and 2,558 genes were detected to specifically respond to drought in the tolerant and the sensitive line, respectively, with a more positive regulation tendency in the tolerant genotype. Furthermore, 4,700, 4,748, 4,403 and 4,288 genes showed differential expression between the two lines under moderate drought, severe drought and their well-watered controls, respectively. Transcription factors were enriched in both genotypic differentially expressed genes and specifically responsive genes of the tolerant line. It was speculated that the genotype-specific response of 20 transcription factors in the tolerance line and the sustained genotypically differential expression of 22 transcription factors might enhance tolerance to drought in maize. Our results provide new insight into maize drought tolerance-related regulation systems and provide gene resources for subsequent studies and drought tolerance improvement.

## Introduction

Maize (*Zea mays* L.) is one of the most important cereal crops worldwide for food and feed [1]. However, due to global climate warming and water scarcity, the frequent occurrences of droughts have become a major threat for maize production [2]. Unfortunately, despite steadily rise of maize yields over the past decades, the sensitivity of maize to drought stress has increased [3]. Therefore, the development of drought-tolerant maize has become an urgent task, and it requires an in-depth understanding of drought responsive molecular regulatory networks and further approaches of genomic selection, genetic engineering or genomic editing of genes responsible for drought tolerance.

The molecular responses of plants to drought stress are complex, involving signal perception, signal transduction and the expression of down-stream regulatory and functional proteins [4]. ABA (abscisic acid) is a classic plant hormone related to drought tolerance, not only as an inducer of stomatal closure but also as a regulator of many drought-inducible genes [5]. Auxin and ethylene are also significantly regulated by drought stress [6, 7]. Their interaction can regulate root development and architecture under drought [8]. Transcription factors (TFs) play important roles in regulating gene expression at the transcriptomic level [9, 10]. Numerous TF genes belonging to different families have been identified to modulate gene expression networks of drought adaption and drought tolerance in plants. TF families such as MYB, MYC, NAC, bZIP, HD-ZIP and DREB have gained broad attentions due to their remarkable roles in plant drought tolerance *via* ABA-dependent or ABA-independent pathways [11]. For example, the expression variations of *ZmDREB2*.*7* were significantly associated with maize drought tolerance at the seedling stage [12]. The overexpression of *ZmNAC111* in transgenic maize yielded enhanced water-use efficiency and increased expression of some drought responsive genes [13]. Heat shock protein (HSP) and peroxidase are typical functional proteins that are responsive to drought. HSPs are molecular chaperons that function in protein folding and assembly, which are involved in the response to various abiotic stresses [14, 15]. Peroxidase catalyzes H_2_O_2_ to OH^-^ and digests the reactive oxygen produced under drought [16].

Different methods and techniques have been used to discover drought responsive genes and elucidate the mechanisms of the responses to drought stress at the transcriptomic level in maize, such as suppression subtractive hybridization (SSH) [17, 18], expression sequence tags (ESTs) [19], and cDNA microarrays [20–24]. Due to the development and reduced cost of next-generation sequencing, RNA-Seq technology has become a powerful method to investigate genome-wide gene expression, including the transcriptomic response to drought stress, e.g. in sorghum [25], rice [26, 27], wheat [28] and soybean [29–31]. In maize, a few reports have used RNA-Seq to analyze global gene expression changes under drought. For example, gene expression profiles of maize fertilized ovary and basal leaf under drought were monitored, and the results showed abundant of decreased expressed genes related to cell division and the cell cycle in the ovary under drought [32]. Moreover the genes related to carbohydrate metabolism, ABA-related processes and phospholipase C-mediated signaling pathway maybe involve in the maize drought response [32]. Opitz *et al*. analyzed the transcriptomic response to low water potential in maize primary roots and reported that the regulated genes in the Gene Ontology (GO) categories “oxidoreductase activity” and “heme binding” were related to the water deficit response to ROS metabolism [33]. Further analysis used a Bayesian hierarchical model to determine the genes’ activity status showed that 1,915 water deficit-responsive genes were conservatively regulated in root tissues, with functional gene categories enriched in transcriptional regulation and hormone metabolism [33].

The drought treatment methods used in most of the previous studies of gene expression profiling under drought stress mainly included simulated drought (in fact osmotic stress) by adding polyethylene glycol (PEG) at the germination or seedling stage [17, 21, 34–37], or soil drought in pots in growth chambers or greenhouses [18–20, 22, 23, 32, 38–40], with sampling tissues after several hours or days of drought treatment. However, these drought treatments have inconformity with the actual drought environment in the field [41]. Thus, it is better to bring drought-related studies back to the field. In fact, very few experiments involving gene expression profiling for drought responsiveness in maize are conducted under soil-drying conditions in the field, except for one example using kernel samples [24].

In this study, two maize inbred lines with contrasting drought tolerance, the tolerant line H082183 and the sensitive line Lv28, were planted in the field under well-watered and natural soil drought treatments. In total, 16 leaf tissue samples from two genotypes, two treatments and two stress levels were used for RNA-sequence analysis. Differential expression analysis between the treatments and genotypes was performed to find drought responsive genes and genotypic differentially expressed genes. The objectives of the present study were to identify candidate genes that might play important roles in the drought-tolerant line and to discover potential genes that may have high breeding values in the genetic improvement of maize drought tolerance. Interestingly, the enrichment of transcription factors in the uniquely drought responsive genes in the tolerant genotype, and in genotypic differentially expressed genes implied that some TFs contribute greatly to drought tolerance and had future breeding potential.

## Materials and methods

### Plant materials and experimental design

Two maize inbred lines were used in this study. Lv28 (L) is an elite inbred line and a representative of the Luda Red Cob heterotic group in China [42]. H082183 (H) is a maize inbred line with an ambiguous pedigree provided by the Maize Research Center of Beijing Academy of Agricultural and Forestry Sciences, which was selected as a drought tolerant inbred line by the agronomic traits under field drought for breeding purposes (unpublished data). The experimental materials were planted in a field at Urumqi in Xinjiang, China (43.98°N, 87.51°E). Two water treatments (well-watered and drought) were set in this study with 5 m apart. In each treatment, a two-row plot was designed for each of the two inbred lines with six replicates. The rows were 3 m length and 0.6 m apart, with the density of about 61,000 plants/ha. For both treatments, the field was drip irrigated for 8 hours at sowing, with same water amount in each row. And then the rows were covered by plastic film to maintain the soil humidity and temperature. When seedlings grew up to V4 (3 weeks after sowing), the film was discarded; then different treatments were started to be applied. The well-watered treatment was irrigated every week (5 hours each time). For drought treatment, watering was withheld till to experiment end (irrigating only once at sowing). Leaf relative water content (RWC) was measured from 9:00 to 11:00 a.m. at 7 time points: 6, 9, 16, 18, 27, 44, 46 days after drought (DAD). The 15 cm apex of last fully expanded leaf was sampled and weighed immediately as fresh weight. Then the leaves were put in water for 24 h at 4°C in darkness and weighted as turgid weight. Dry weight was measured after placing the leaves at 103°C for 0.5 h and 72°C for 24 h. The formula of RWC is (fresh weight–dry weight) / (turgid weight–dry weight) × 100% [43]. The 15 cm apex of second last fully expanded leaf was sampled for sequencing at the same time. The sequencing samples were quickly frozen in liquid nitrogen after sampling and stored at -80°C.

Based on leaf RWC results, we selected the samples at 27 DAD as moderate drought (MD, RWC = 84–90%) and 46 DAD as severe drought (SD, RWC = 82–84%) for sequencing, along with the well-watered controls of moderate drought (moderate drought control, named MC) and severe drought (severe drought control, named SC). For each genotype and treatment, two individual plant leaf samples with the closest RWC from six replicates were chosen as two biological replicates for sequencing. In total, 16 leaf samples of 2 genotypes (H, L) x 2 treatments (well-watered and drought stress) x 2 stress level (moderate drought and severe drought) x 2 biological replicates were sequenced.

### RNA extracted and library construction

Total RNA of leaf samples was extracted using TRIzol reagent (Invitrogen, CA, USA). The RNA degradation and contamination were checked on 1% agarose gels. The RNA quality and integrity were assessed *via* Nanodrop 2000 Spectrophotometer (Thermo Fisher Scientific, Wilmington, DE) and Bioanalyzer 2100 using RNA Nano 6000 Assay Kit (Agilent Technologies, CA, USA). The RNA concentration was measured using Qubit RNA Assay Kit in Qubit 2.0 Fluorometer (Life Technologies, CA, USA). Construction of cDNA libraries was performed using NEBNext Ultra RNA Library Prep Kit for Illumina (NEB, MA, USA). Briefly, mRNA was attached to magnetic beads with oligo(dT), and then cleaved into short fragments using divalent cations under elevated temperature. Then the first strand of cDNA was synthesized using random hexamer primers. Subsequently, second strand cDNA synthesis was performed using DNA polymerase I and RNase H, and then cDNA was ligated with adaptors. These fragments were purified and used as templates for PCR amplification to construct the sequencing cDNA libraries.

### Sequencing data

The raw sequencing data were produced on the Illumina HiSeq 2000 (Illumina, CA, USA). Reads containing adapter or low-quality reads (e.g. reads containing poly-N, containing more than 10% unknown base, or with greater than 50% bases whose Q_phred_ lower than 20) were removed to obtain clean reads for the next analysis. The maize reference genome B73_RefGen_v3 sequence and gene annotation files were downloaded from ftp://ftp.ensemblgenomes.org/pub/plants/release-24/fasta/zea_mays/ and ftp://ftp.ensemblgenomes.org/pub/plants/release-24/gtf/zea_mays/. Tophat2 [44] software was used to align clean reads to the reference genome with default parameters. The aligned reads were assembled by Cufflinks [45]. Cuffdiff was used to calculate the FPKM (fragments per kilo base of transcript per million fragments mapped) of each gene. Hierarchical clustering analysis of samples transcripomes was performed using the log_2_(FPKM + 1) value of the genes in each library. PCA analysis of sampels’ transcriptomes was conducted and plotted by DESeq [46] package in R software.

### Differential expression analysis

To identify genes with different expression levels, a pairwise comparison algorithm of DESeq [46] was used. Briefly, for each library, the Sequence Alignment/Map (SAM) files created by Tophat2 were used to calculate mapped reads of the genes by HTSeq [47]. Then the read counts were used for genes expression normalization by DESeq based on the negative binomial distribution model. In addition, the *P* value of the results was adjusted using the Benjamini and Hochberg’s approach to control the false discovery rate (FDR). Two comparison assays were designed. The first assay compared gene expression under drought and well-watered environments of the same genotype to detect drought responsive genes, including four comparison groups: HMD-HMC, LMD-LMC, HSD-HSC and LSD-LSC. The second assay compared genes expression of the different genotypes under the same environment to detect genotypic differentially expressed genes, including four comparison groups: HMD-LMD, HMC-LMC, HSD-LSD and HSC-LSC.

### Gene function annotation analysis

Gene Ontology analysis was conducted by agriGO (http://bioinfo.cau.edu.cn/agriGO/) [48], using Singular Enrichment Analysis (SEA) with *Zea mays* AGPv3.30 as the reference genome background. Significantly enriched GO terms were determined by FDR < 0.05 with the Fisher statistical test and the Bonferroni multi-test adjustment. For the stress-related genes (with GO categories “response to stimulus”, “response to stress”, “response to abiotic stimulus” and “response to endogenous stimulus”), further function analysis was performed by blasting to the Swiss-Prot (http://www.uniprot.org/) [49] database with default parameters for validating the known functions or annotations of orthologous genes. The enrichment of transcription factors was tested using Fisher’s exact test. All 3,308 transcription factor genes included in PlantTFDB (http://planttfdb.cbi.pku.edu.cn/) [50] were used as a genome-wide TFs background gene set and 38,136 genes were used as the whole maize genome background.

### Quantitative real-time RT-PCR

Eight genes were randomly selected to monitor their expression using quantitative real-time RT-PCR (qRT-PCR). The first strands of the cDNA fragments were synthesized from total RNA using TransScript One-Step gDNA Removal and cDNA Synthesis SuperMix (TransGen Biotech, Beijing, China). The qRT-PCR was performed on Real-time fluorescent quantitative PCR instrument ABI7500 (Applied Biosystems, CA, USA). Each 20 μl PCR contained 0.8 μl cDNA, 10 μl 2× SYBR premix Ex Taq (Takara, Japan), 8 μl ddH_2_O, 0.4 μl Dye II, 0.4 μl forward primer and 0.4 μl reverse primer. The thermal cycling conditions were as follows: 95°C for 30 s, 40 cycles of 95°C denaturation for 5 s and 60°C annealing for 34 s. The maize *GAPDH* gene was used as the internal control. Each sample was repeated three times. The relative expression levels were calculated by the 2^-ΔΔCt^ method [51]. The primer sequences are listed in **S12 Table**.

## Results

### Phenotypic analysis of drought tolerance

**Fig 1:**
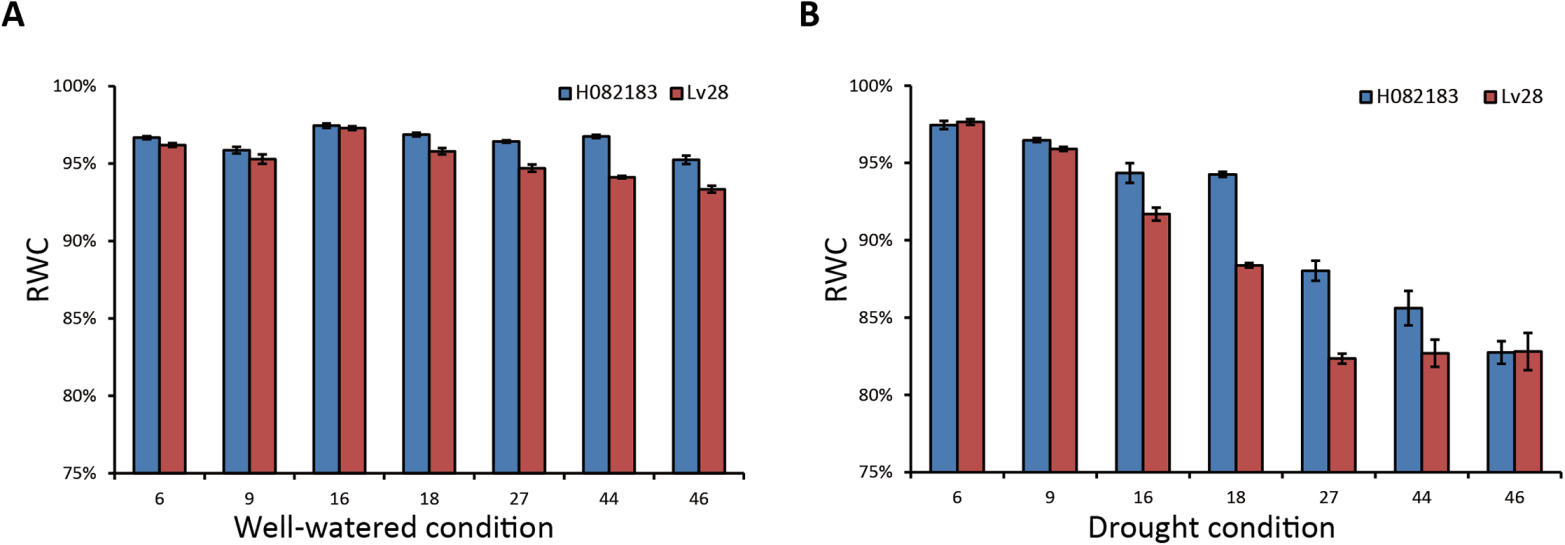
Change in the relative water content in each line. The relative water content (RWC) of the leaves was measured under well-watered (**A**) and water stress (**B**) conditions.

Two maize inbred lines H082183 (H) and Lv28 (L) were used in this study. To analyze the phenotypic response to drought, the leaf RWC of each genotype under drought and well-watered treatments was monitored at 7 time points after the treatment began (**Fig 1**). Under drought, the leaf RWCs of both lines decreased from 96–98% to 82–84% when the drought lasted, and the RWCs of the two genotypes under drought were significantly (*P* < 0.01) lower than their well-watered controls from 16 days after drought (DAD) to 46 DAD (**S1 Table**). Meanwhile, under drought condition, the RWCs of H082183 were significantly higher than those of Lv28 during 9–44 DAD (*P* < 0.01) (**Table 1**). However, the RWCs of H082183 and Lv28 both reached 82–84% at 46 DAD.

**Table 1:** Statistical analysis of the relative water content of the different genotypes under drought.

DAD^a^	H082183		Lv28		*p*-value^b^
	Mean	±SD	Mean	±SD	
6	97.45%	0.26%	97.66%	0.19%	< 0.05
9	96.47%	0.13%	95.90%	0.14%	< 0.01
16	94.35%	0.64%	91.69%	0.42%	< 0.01
18	94.26%	0.17%	88.38%	0.15%	< 0.01
27	88.03%	0.65%	82.35%	0.32%	< 0.01
44	85.62%	1.11%	82.69%	0.87%	< 0.01
46	82.74%	0.74%	82.80%	1.20%	> 0.05

^a^ Days After Drought.

^b^ *p*-value: Student’s t-test.

### Sequencing and mapping results

After removing the adapter sequences and reads with low quality of raw RNA-Seq data, 1.6 billion paired-end clean reads were obtained, with 101.7 million reads per library on average (**S2 Table**). All clean sequencing data were deposited in NCBI Sequence Read Archive (SRA, https://www.ncbi.nlm.nih.gov/sra) under accession number SRP102142. The Q30 score of all libraries were above 94%, with a mean of 95.2%, indicating reliable results of sequencing. On average, 85.2% and 85.1% reads were mapped to the reference genome in H082183 and Lv28, respectively, with Tophat2 default parameters (**S3 Table**). Among these mapped reads, 55.5% and 55.1% reads in H082183 and Lv28 were uniquely mapped, respectively.

### Gene expression profiles of each library

**Fig 2:**
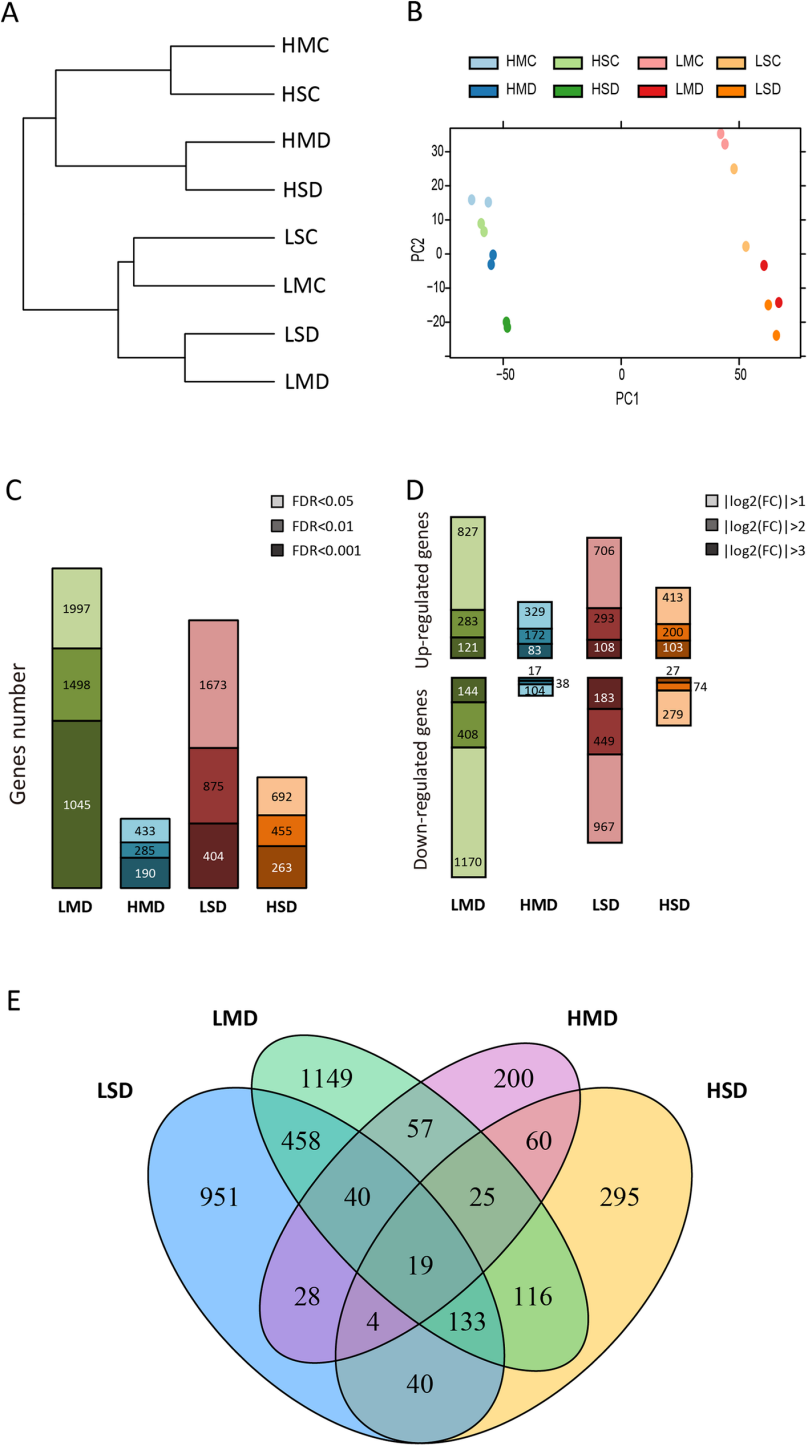
The relation of samples transcriptome and the drought responsive genes. (**A**) Hierarchical clustering was performed using the log_2_(FPKM + 1) of the samples. H and L present H082183 and Lv28, respectively. MD and SD present moderate drought and severe drought, respectively. MC and SC present well-watered controls of moderate drought and severe drought, respectively. (**B**) PCA analysis of the sample transcriptomes. (**C**) Drought responsive gene numbers of the two genotypes under moderate and severe drought with different FDR criteria (|log_2_(fold change)| > 1). (**D**) Number of up- and down-regulated drought responsive genes with different fold change levels (FDR < 0.05). (**E**) The overlap of the drought responsive genes of the two genotypes under two drought conditions.

Genome-wide gene expression was estimated with fragments per kilo base of transcript per million fragments mapped (FPKM) by using Cufflinks [45]. The results of correlation analysis revealed that the transcriptomes of the two biological replicates for each genotype and condition were significantly correlated (*r*> 0.95, *p*<0.01) (**S1 Fig**). Hierarchical clustering revealed that the samples of each genotype were clustered together according to the transcriptome data (**Fig 2A**). And then, the samples of the different water treatments (drought and well-watered control) were separated in both of the two genotypes. The PCA plot demonstrated that the samples of the different genotypes were distantly divided into two groups, and the sensitive line’ samples were more dispersive (**Fig 2B**). These results indicated that the transcriptomes of the two genotypes were strongly affected by drought.

### Differentially expressed genes from treatments comparing assays

To identify genes responsive to drought in both genotypes, differential expression analysis was performed within four comparing groups: HMD-HMC, LMD-LMC, HSD-HSC and LSD-LSC. In total, 1,997 and 1,637 differentially expressed genes (DEGs) were detected respond to drought under MD and SD, respectively, in Lv28 (|log_2_(fold change)| > 1, FDR < 0.05) (**Fig 2C**). However, only 433 and 692 genes were differentially expressed in the drought-tolerant line H082183 under MD and SD, respectively. The order of the DEG number in four comparison assays was constant within different criteria (FDR < 0.05, < 0.01, < 0.001): LMD > LSD > HSD > HMD. Among the drought responsive genes, the down-regulated gene counts were greater than the up-regulated gene counts within different fold change thresholds (|log_2_(fold change)| > 1, > 2, > 3) in Lv28 under both drought environments, while opposite results were obtained in H082183 (**Fig 2D**).

Based on the overlapping of DEG (|log_2_(fold change)| > 1, FDR < 0.05) sets acquired by different comparison assays, drought responsive genes could be classified into two groups: (I) genotype-specific responsive genes and (II) common drought responsive genes shared by both genotypes (**Fig 2E**). For the genotype-specific responsive genes, there were 555 genes (200 genes in MD, 295 genes in SD and 60 genes in both MD and SD) uniquely responded to drought in tolerant line H082183. In addition, 2,558 genes (1,149 genes in MD, 951 genes in SD and 458 genes in both MD and SD) were specifically responded to drought in sensitive line Lv28. Other than these genotype-specific drought responsive genes, there were 462 common drought responsive genes in H082183 and Lv28 under drought.

### Genotype-specific drought responsive genes

**Fig 3:**
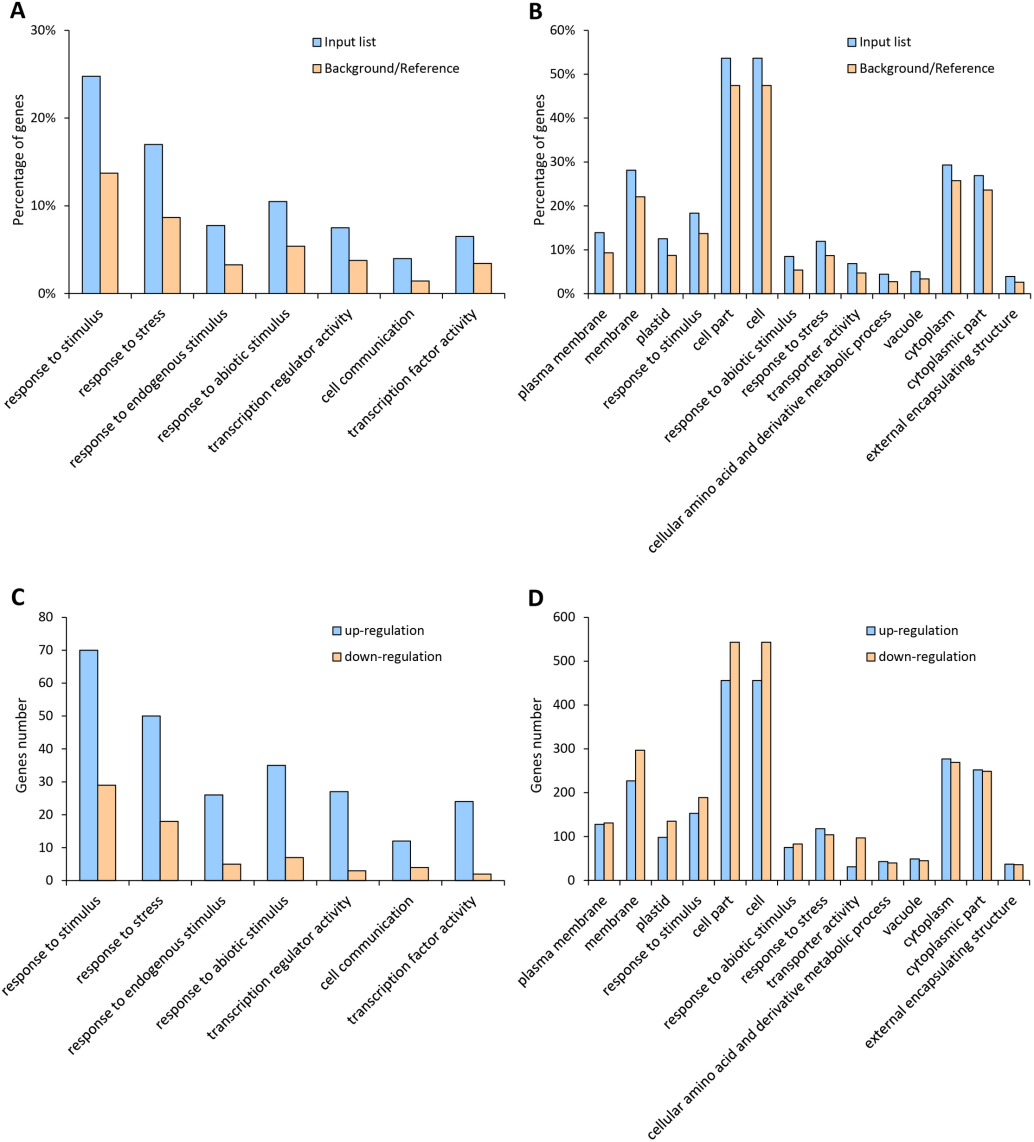
Gene Ontology analysis of genotype-specific responsive genes under drought. GO enrichment of genotype-specific DEGs under drought in H082183 (**A**) and Lv28 (**B**). The numbers of up-regulated and down-regulated genes of each category in H082183 (**C**) and Lv28 (**D**) were shown.

GO annotation and enrichment analysis were performed within the genotype-specific drought responsive genes. For the tolerant line H082183, the unique drought responsive genes were enriched in biological process GO terms “response to stimulus”, “response to stress”, “response to endogenous stimulus”, “response to abiotic stimulus”, “cell communication” and molecular function GO terms “transcription regulator activity”, “transcription factor activity” (**Fig 3A**). Meanwhile, in the sensitive line Lv28, the responsive genes were enriched in cellular component GO terms “plasma membrane”, “membrane”, “plastid”, “cell part”, “cell”, “vacuole”, “cytoplasm”, “cytoplasmic part”, “external encapsulating structure”, biological process GO terms “response to stimulus”, “response to abiotic stimulus”, “response to stress”, “cellular amino acid and derivative metabolic process” and molecular function GO terms “transporter activity” (**Fig 3B**). These results indicated that the tolerant line specific responsive genes were involved in signal transduction pathways or regulation systems responsive to drought. We further analyzed the regulation patterns of the genes under each enriched GO term in both genotypes. Interestingly, the enriched responsive genes showed an extraordinarily different regulation tendency between the drought tolerant line and the drought sensitive line (**Fig 3C and 3D**). In H082183, 70.71%, 73.53%, 83.87%, 83.33%, 90.00%, 75.00% and 92.31% of the drought responsive genes showed up-regulated under drought in the GO terms “response to stimulus”, “response to stress”, “response to endogenous stimulus”, “response to abiotic stimulus”, “cell communication” and “transcription factor activity”, respectively. However, this regulation situation was not observed in the Lv28-specific drought responsive genes, with an approximately equal number of up-regulated and down-regulated genes under each GO term.

### Common drought responsive genes for both genotypes

There were 462 common drought responsive genes for tolerant line H082183 and sensitive line Lv28 (**S4 Table**). The functions of these common genes were mostly related to stress response (**Table 2**). Among the 462 common drought responsive genes, the expression patterns (up- or down-regulation) of 449 genes were concordant between the two lines under drought. These results revealed the existence of conservative drought-induced regulation pathways between the two genotypes. Furthermore, there were 19 drought responsive genes commonly shared by H082183 and Lv28 under both moderate and severe drought, indicating that the signaling pathways or regulation system they are involved in may be absolutely necessary for plants responding to drought stress (**S5 Table**). Among the 19 genes, seven genes were down-regulated, including an AP2/EREBP gene, a DELLA protein and some other unknown genes. The up-regulated genes were mainly responsive to water deprivation, abscisic acid and ethylene or the regulation of transcription.

**Table 2:** GO enrichment analysis of common drought responsive genes between the two maize inbred lines with contrasting drought tolerance.

GO term	Ontology^a^	Description	No. of genes	*p*-value	FDR
GO:0006950	P	response to stress	36	7.20E-09	4.10E-07
GO:0009628	P	response to abiotic stimulus	27	2.00E-08	1.20E-06
GO:0050896	P	response to stimulus	43	5.70E-07	3.20E-05

^a^ P: Biological Process

However, 13 genes showed opposite expression patterns in response to drought between the tolerant and sensitive lines (**S6 Table**). Among these genes, 10 genes were regulated oppositely under moderate drought, with eight genes up-regulated in H082183 while down-regulated in Lv28. These genes included an AP2/EREBP transcription factor superfamily gene (GRMZM2G438202), an auxin-responsive SAUR family gene (GRMZM2G447151), a gene encoding pentatricopeptide repeat containing protein (GRMZM2G016866), a LURP-one-related gene (GRMZM2G145655), an adenine phosphoribosyltransferase gene (GRMZM2G179810), and two formin-like protein genes (GRMZM2G067830, GRMZM2G360234). A cytidine triphosphate (CTP) synthase gene (GRMZM2G132547) and a cytochrome P450 protein (GRMZM2G065635) were up-regulated in Lv28 but down-regulated in H082183 under moderate drought. Moreover, three genes had opposite expression patterns under severe drought stress between H082183 and Lv28. Two genes were up-regulated in Lv28 but down-regulated in H082183, with a gene encoding an H/ACA ribonucleoprotein complex subunit 4 protein (GRMZM2G044128) and a gene encoding a guanine nucleotide-binding protein (GRMZM2G429113). The other gene encoding a WAT1-related protein (GRMZM2G030216) was up-regulated in H082183 but down-regulated in Lv28 under severe drought.

### Differentially expressed genes in genotype comparison assays

**Fig 4:**
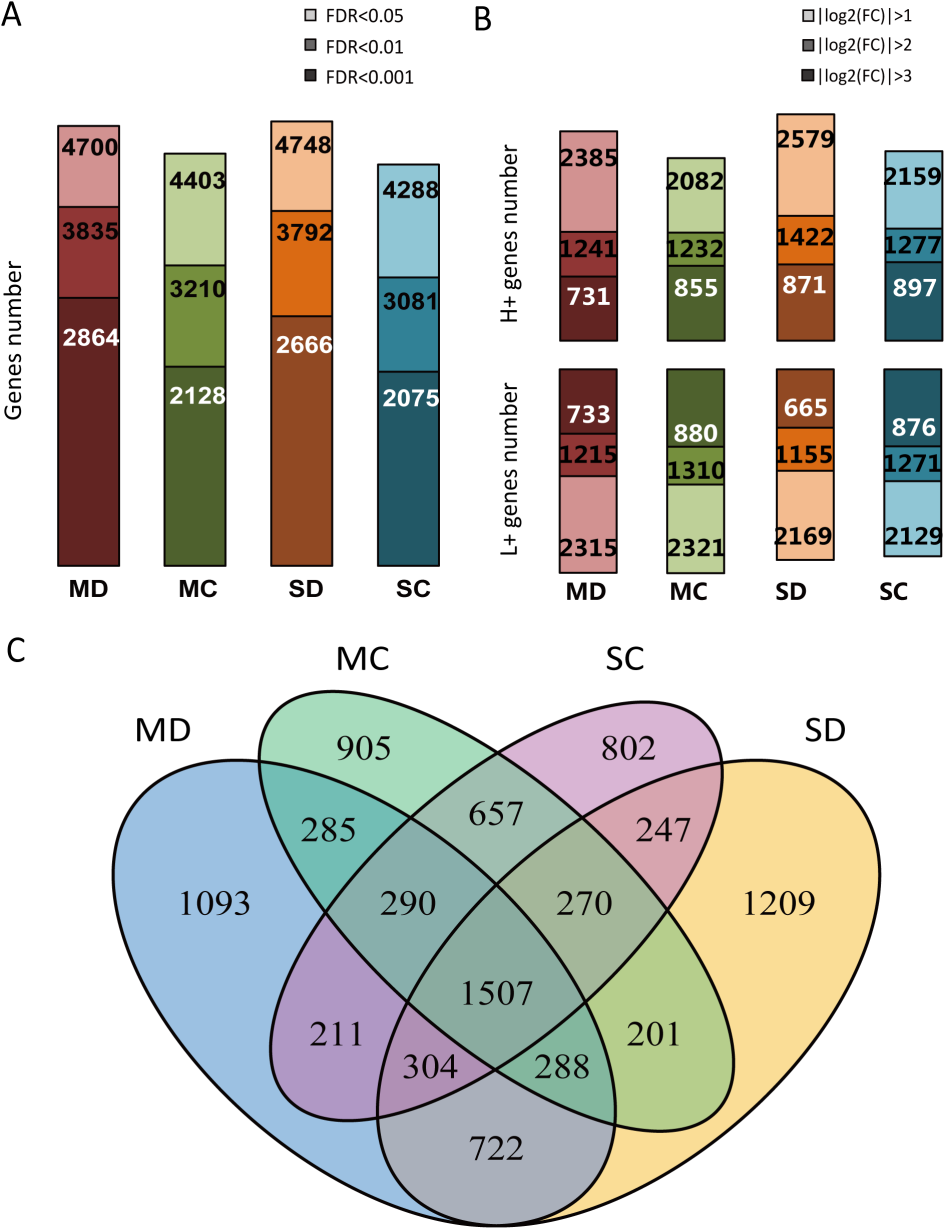
The numbers and overlap of genotypic DEGs. (**A**) Genotypic DEGs numbers under moderate drought (MD), severe drought (SD), moderate drought control (MC) and severe drought control (SC) with different FDR criteria (|log_2_(fold change)| > 1). (**B**) The numbers of H082183 up-regulated (H+) and Lv28 up-regulated (L+) DEGs under four conditions with different fold change levels (FDR < 0.05). (**C**) The overlap of genotypic DEGs under different conditions.

DEG analysis was also performed to discover the genetic difference between the tolerant and the sensitive genotypes under different water conditions. Four comparison groups of H082183 and Lv28 under drought and well-watered controls were performed: HMD-LMD, HMC-LMC, HSD-LSD and HSC-LSC. The results showed that 4,700, 4,748, 4,403 and 4,288 genes were differentially expressed in the comparing groups of HMD-LMD, HMC-LMC, HSD-LSD and HSC-LSC, respectively (**Fig 4A**). The numbers of the tolerant line up-regulated (H+) genes showed little difference with the sensitive line up-regulated (L+) gene number in different thresholds (|log_2_(fold change)| > 1, > 2, > 3) (**Fig 4B**). Among the 4,700 DEGs in moderate drought, 2,385 genes showed higher expression in H082183 (H+), and 2,315 genes were more significantly induced in Lv28 (L+). Under severe stress, the H+ and L+ genes were 2,579 and 2,169, respectively. In total, 1,507 genes were commonly differentially expressed in all conditions (**Fig 4C**). Among the 1,507 commonly differentially expressed genes, the expression patterns (H+ or L+) of 1,501 genes were the same in four comparing assays, including 806 H+ regulated genes and 695 L+ regulated genes, respectively (**S7 Table**). A total of 3,024 genes (1,093 genes in moderate drought, 1,209 genes in severe drought and 722 genes in both moderate and severe drought) were uniquely differentially expressed under drought between the tolerant and sensitive lines.

**Fig 5:**
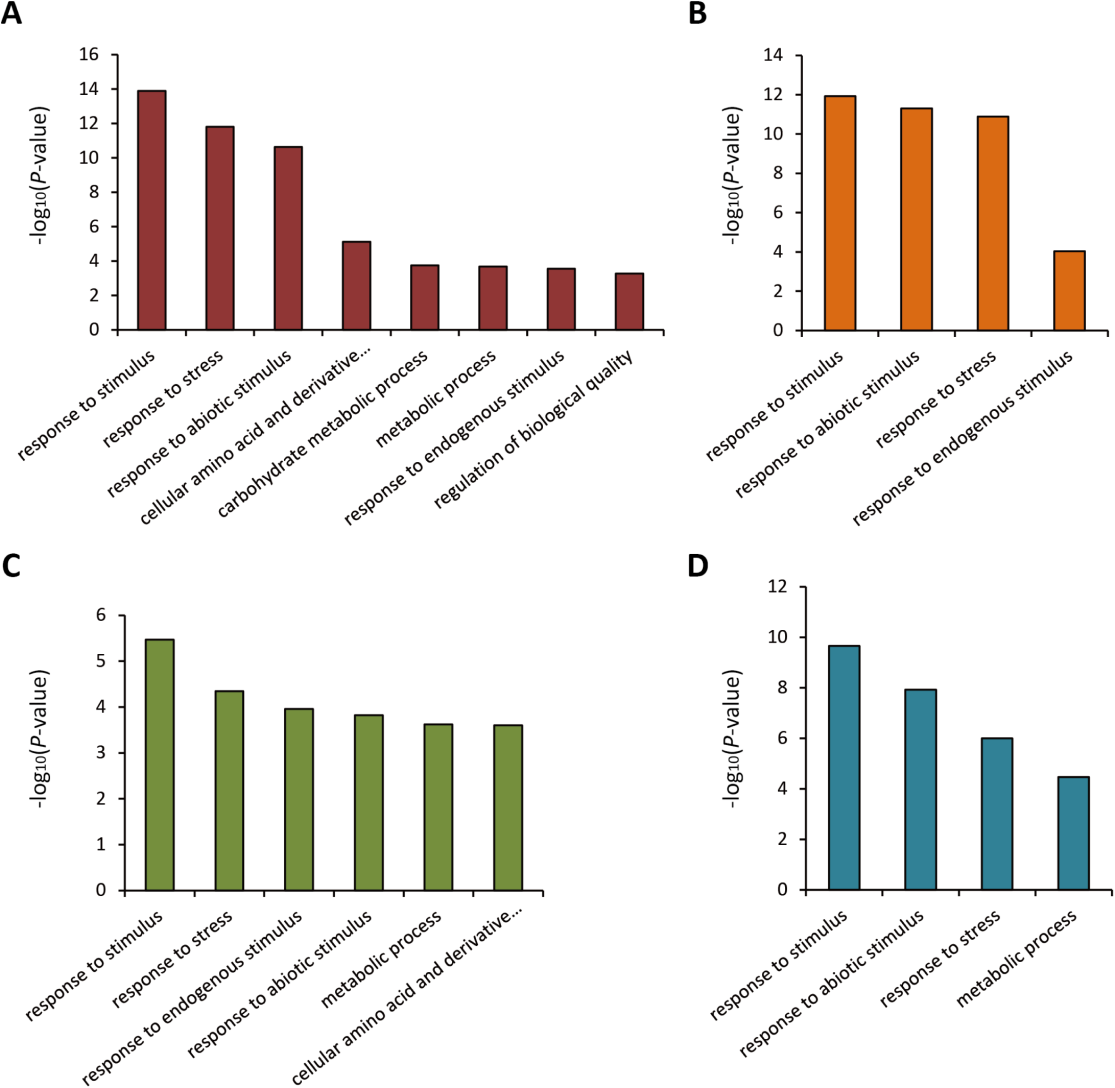
GO enrichment analysis of genotypic DEGs. The significant (FDR < 0.05) enriched GO terms and the–log_10_(*P*-value) under moderate drought (**A**), severe drought (**B**), moderate drought control (**C**) and severe drought control (**D**) were plotted.

To understand the function of genotypic DEGs under each drought condition, GO enrichment analysis of DEGs obtained from four comparing groups were performed. The results showed that the most significant enriched GO terms of genotypic DEGs from all four comparing assays were “response to stress” and “response to stimulus” (**Fig 5**). These results revealed that the constantly differential expression of stress-related genes between the tolerant and the sensitive lines under drought or normal water conditions probably caused the different drought tolerance performances. In total, 612, 522, 480 and 495 drought-related genes under moderate drought (MD), severe drought (SD), moderate drought control (MC) and severe drought control (SC) were selected according to their GO annotation (“response to stress”, “response to stimulus”, “response to abiotic stimulus” or “response to endogenous stimulus”) (**S8 Table**). Among these genes, 268, 244, 210 and 241 genes were up-regulated in the drought tolerant line under MD, SD, MC and SC, respectively, while 344, 278, 270 and 254 genes were up-regulated in the sensitive line under MD, SD, MC and SC, respectively (**Table 3**). There were 312 stress-related DEGs under both moderate drought and severe drought, with 136 genes differentially expressed under all four conditions (**S2 Fig**). Further gene function analysis of these 312 genes was performed with the Swiss-Prot database. The results showed that 22 genes were transcription factors, including 13 auxin response factors (ARFs), and 21 heat shock proteins (HSPs) were contained in the 312 stress-related genes (**S9 Table**).

**Table 3:** The numbers of H082183 up-regulated and Lv28 up-regulated stress-related DEGs.

Condition	H+ genes number ^a^	L+ genes number ^b^
Moderate Drought	268	344
Severe Drought	244	278
Moderate drought Control	210	270
Severe drought Control	241	254

^a^ H+ represents H082183 up-regulated genes.

^b^ L+ represents Lv28 up-regulated genes.

### Important transcription factors in drought tolerance

**Fig 6:**
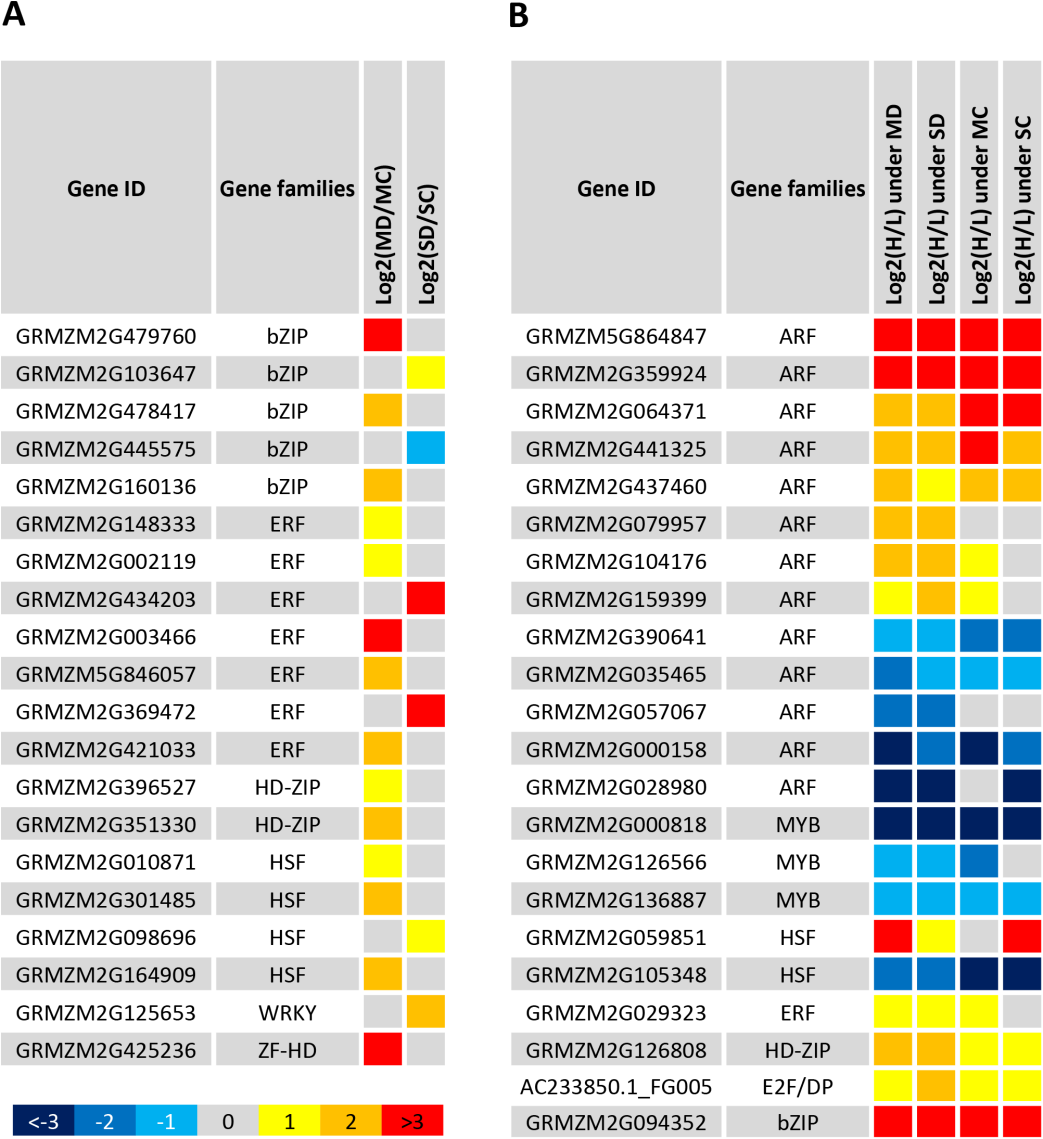
Transcription factors (TF) enriched in H082183, the drought tolerant line. The TFs are listed with their log_2_(Fold change). (**A**) Twenty TF genes were enriched in H082183-specific responsive genes, including seven ERF genes and five bZIP genes. Most of them showed an active regulation pattern under drought. (**B**) Twenty two TF genes were enriched in genotypic DEGs between the tolerant and the sensitive lines under drought, including thirteen ARF genes. H and L present H082183 and Lv28, respectively. MD and SD present moderate drought and severe drought, respectively. MC and SC present moderate drought control and severe drought control, respectively. The different color represented different level of log_2_(fold change).

Twenty TF genes were enriched in the genotype-specific drought responsive genes (**Fig 3A**, in the enriched GO term “transcription regulatory activity”; **Fig 6A**; **S10 Table**). For example, seven ERF genes were specifically responsive to drought in the drought tolerant line. GRMZM2G369472 is the orthologous gene of AT4G34410 encoding AtERF109. GRMZM2G434203 was up-regulated under severe drought (with log_2_FC>4), and its orthologous gene AT1G12630 encodes ERF027. GRMZM2G148333, GRMZM2G002119, GRMZM5G846057 and GRMZM2G421033 were up-regulated under moderate drought and their orthologs, AT1G53910, AT1G28360, AT1G64380 and AT1G46768 encode RAP2-12, ERF16, ERF061 and RAP2-1, respectively. Five of the seven ERF genes were responsive to moderate drought, suggesting that the specific up-regulation of these ERF genes might enhance the tolerance or adaption to drought in maize. Five bZIP TFs showed responsive to drought in the tolerant line, with one gene GRMZM2G479760 enormously up-regulated under moderate drought, whose orthologous gene encodes an abscisic acid-insensitive 5-like protein. However, GRMZM2G445575, whose orthologous gene encodes TGA3, was down-regulated under drought. Four HSF genes were up-regulated in tolerant genotype under drought, including GRMZM2G010871 (ortholog of *HSFA6B*), GRMZM2G301485 (ortholog of *HSFB3*), GRMZM2G098696 (ortholog of *HSFB2B*) and GRMZM2G164909 (ortholog of *HSFB2B*). Notably, the most specific responsive TFs were up-regulated compared with the well-watered control.

Twenty-two TF genes were enriched in the 312 stress-related genotypic DEGs under both moderate drought and severe drought (**S9 Table; Fig 6B**). Of these genes, 13 genes were auxin response factors (ARF). Among the 13 ARF genes, eight genes were up-regulated in the drought tolerant line under drought. GRMZM2G864847 and GRMZM2G359924, which were orthologs of *OsIAA16*, were up-regulated more than 8-fold in the tolerant genotype under drought and well-watered controls. However, 5 ARF genes were down-regulated under drought in the tolerant line compared with the sensitive line, including GRMZM2G028980 (ortholog of *OsARF6*), which was down-regulated greater than 8-fold under drought. A bZIP transcription factor gene GRMZM2G094352 was up-regulated in the tolerant line under all four conditions. Three MYB transcription factor genes were down-regulated in the tolerant line, with GRMZM2G000818 (*MYB1*) down-regulated under drought and normal water conditions. To validate the enrichment of transcription factors in all responsive genes or genetic DEGs, a Fisher exact test of these 42 genes was performed using all TFs expressed in the maize genome as the background. The 20 and 22 important TF genes were both significantly enriched compared with their background gene sets (**S11 Table**).

### Validation of DEGs by qRT-PCR

**Fig 7:**
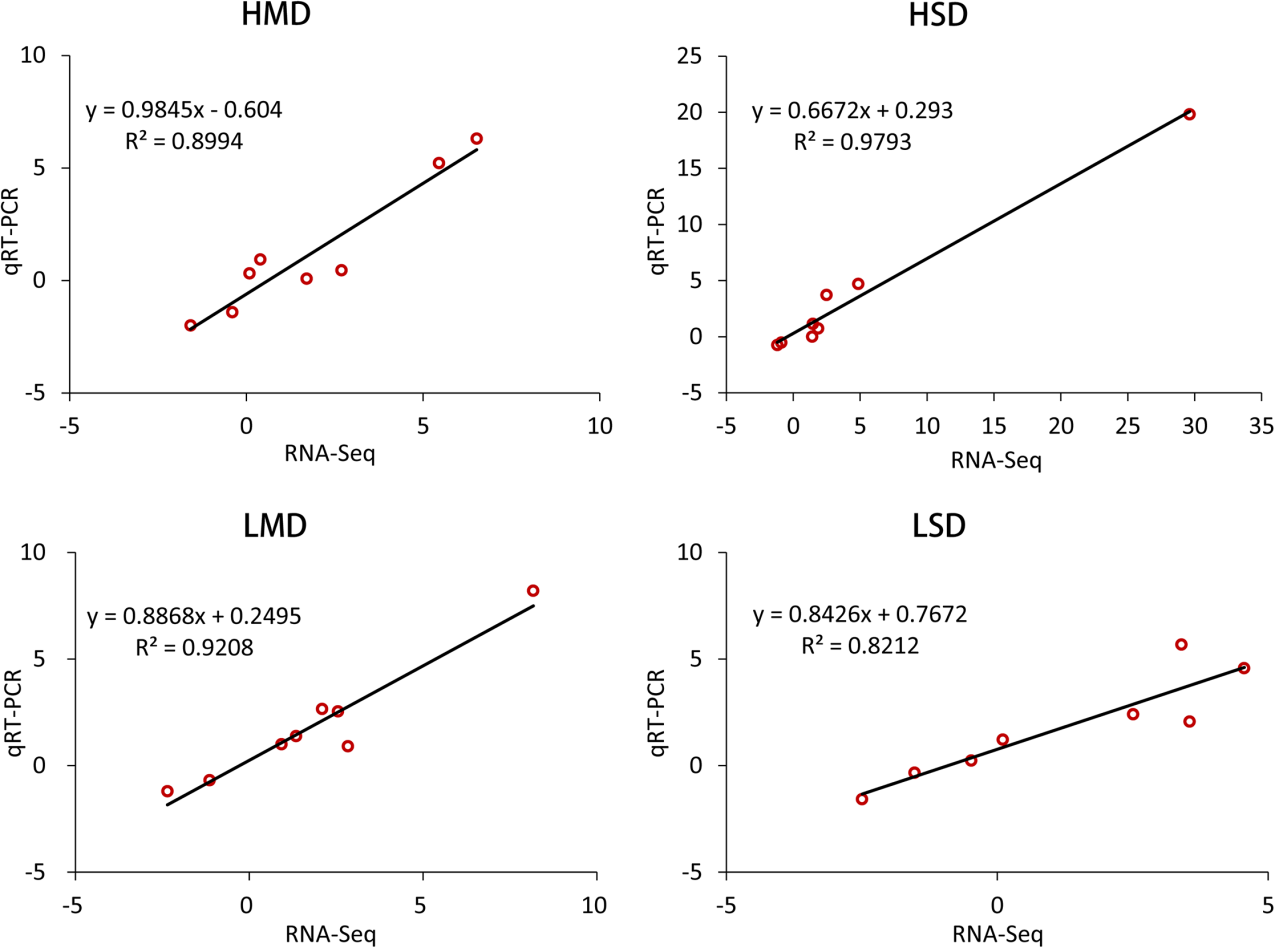
Validation of DEGs by qRT-PCR and RNA-Seq. Eight genes were selected to perform qRT-PCR validation in both moderate drought (MD), severe drought (SD) and their well-watered controls (MC, SC) of H082183 and Lv28.

To confirm the expression patterns of the DEGs detected by RNA-Seq, quantitative real-time PCR (qRT-PCR) experiment was performed with eight randomly selected DEGs in H082183 and Lv28. The gene expression was also converted into log_2_(fold change), which was used to analyze the DEGs. The qRT-PCR results showed a high similarity of gene expression trends (up- or down- regulation) with the RNA-Seq data (**S3 Fig**). The only inconformity in the gene GRMZM2G154735 in Lv28 under severe drought may be caused by the little expression change under drought and well-watered conditions. Correlation analysis of the gene expression change obtained by qRT-PCR and RNA-Seq showed a significant correlation (R^2^ > 0.8) in H082183 and Lv28 under both moderate and severe drought (**Fig 7**).

## Discussion

The drought tolerance of plants is a complex process, with responses at different level. At the cellular and molecular level, a range of genes are involved in the adaption and response to drought. These genes can be divided into two groups: functional protein genes and regulatory genes [51]. The functional proteins include late embryogenesis abundant (LEA) proteins, transporters, detoxification enzymes and osmolyte biosynthesis enzymes [5]. For regulatory genes, a regulation network module of stress perception, signal transduction and functional gene regulation has been established [52].

Genome-wide gene expression analysis by RNA-Seq provides a powerful method to monitor the transcriptomic status and mine drought tolerance-related genes in maize [32, 33, 37]. In our study, numerous genes were identified responsive to drought or showed genotypic differential expression between the two lines with contrasting drought tolerance. The contrary gene regulation tendency responded to drought and diverse gene function enrichment between the drought tolerant and the sensitive lines provided a transcriptomic view of the drought-related regulatory network in maize. In general, the overrepresentation of transcription factors (TFs) in the tolerant line specifically responsive genes and genotypic differentially expressed genes suggested a diverse drought tolerance mechanism in maize.

### Drought-related transcription factors

Transcription factors (TFs) play an irreplaceable role in mediating signal transduction in the plant response to drought stress [51]. MYB/MYC, WRKY, bZIP, DREB (AP2/ERF) and NAC were well-known transcription factor families responsive to drought [9]. Several TFs were also responsive to drought stress in the present study.

DREB (dehydration responsive element binding proteins) and ERF (ethylene response factor) are two subgroups of the AP2/ERF (Apetala 2/Ethylene Response Factor) transcription factor families. DREB TFs regulated stress-related genes’ expression in an ABA-independent manner, by interacting with DRE (dehydration responsive element) sequences [53]. Overexpression of *AtDREB2A* could induce drought-, salt- and heat-responsive genes in *Arabidopsis* [54]. A rice DREB gene, *OsDREB2A*, could be induced by drought and improved the survival rates under drought by overexpression [55]. *ZmDREB2A* was induced by dehydration and heat in maize seedlings, whose inducible or constitutive overexpression showed improved drought tolerance [56]. ERF genes are involved in the response to abiotic stress and are considered as candidate genes for stress tolerance engineering in plants [57]. Two specific responsive ERF genes in H082183 have been cloned and proved to be related to drought tolerance. GRMZM2G380377 (*ZmDBP4*) functioned *via* binding to dehydration responsive elements and could improve the tolerance to drought and cold stress when overexpressed in *Arabidopsis* [58]. GRMZM2G061487 (*ZmDBF1*) regulated the expression of the ABA-responsive gene *rab17* in an ABA-dependent pathway [59].

ARFs (auxin response factors) and AUX/IAAs are indispensable for the auxin signaling pathway, which regulates plant growth and developmental processes [60]. The interaction of ARFs and AUX/IAAs could regulate the downstream auxin responsive genes and contribute to drought tolerance [61]. For instance, *OsIAA6*, was highly induced by drought, and its overexpression in transgenic rice showed improved drought tolerance [62]. However, the auxin signal pathway has not gained much attention as a drought-related regulation participant. In our study, 13 ARF genes were listed in the stress-related genotypic DEGs, suggesting their important roles in maintaining relative normal growth or development under drought in maize.

NAC TFs are also involved in the regulation of drought-related genes as transcriptional activators or repressors [63]. The overexpression of *SbSNAC1*, a sorghum NAC gene, conferred improved drought tolerance in transgenic *Arabidopsis* [64]. The maize NAC gene, *ZmSNAC1*, was cloned and characterized could be induced by drought and other stresses, and its overexpression in transgenic *Arabidopsis* yielded enhanced dehydration tolerance [65]. In this study, one NAC gene, GRMZM2G127379 (*ZmNAC111*), was up-regulated in H082183 under severe drought. In maize, an insertion of a miniature inverted-repeat transposable element (MITE) in the promoter region of *ZmNAC111* was significantly associated with maize drought tolerance [13]. Its orthologous gene in rice was proved could enhanced tolerance to drought when overexpressed [66].

MYB TFs have been identified to participate in the drought response in *Arabidopsis* and some other crops by regulating stomatal movement and cuticular wax synthesis [67]. *OsMYB3R-2* was a drought-inducible R1R2R3 MYB transcription factor in rice, whose overexpression in transgenic *Arabidopsis* conferred enhanced drought tolerance [68]. Wheat *TaMYBsm1* gene encodes a R2R3 type MYB protein, whose overexpression in transgenic *Arabidopsis* yielded higher germination rates under drought [69]. However, three MYB transcription factor genes were down-regulated in the tolerant line compared with the sensitive line under drought, suggesting that complex regulatory networks were involved in the drought response of different genotypes.

### Heat shock proteins in stress adaption

Heat shock proteins (HSPs) are molecular chaperones that play key roles in maintaining protein folding, assembly, translocation and stabilizing membranes under stress conditions [70]. Various HSPs have been studied and identified related to multiple stresses. The expression of *AtHSP17*.*6A* was induced by heat and osmotic stress, and the overexpressed plants showed enhanced tolerance to salt and drought [71]. The overexpression of *sHSP17*.*7* in rice increased the survival rates under drought and enhanced heat tolerance and UV-B resistance [72, 73]. However, the overexpression of *AtHsp90*.*2*, *AtHsp90*.*5* and *AtHsp90*.*7* in *Arabidopsis* increased the plant sensitivity to drought and salt stresses, but improved the tolerance to Ca^2+^ concentrations [74]. In this study, 21 heat shock proteins (HSPs) were genotypically differentially expressed between the two lines under drought (**S9 Table**). For example, GRMZM2G158232 is the ortholog of *OsHSP17*.*0* in rice, which can enhance the tolerance to drought and salt stresses [75]. GRMZM2G007729 is the orthologous gene of *OsHSP24*.*1*, whose expression is enhanced by PEG treatment but suppressed by ABA [76].

### Drought-related genes

Numerous studies have identified drought responsive genes using different methods at the transcriptome level. For example, the transcriptomic analysis using a Maize Genome Array of two maize inbred lines under drought in pots revealed more responsive genes in the sensitive line, and cell wall-related genes and transporter genes were differentially expressed between the two lines under drought [22]. The transcriptomes of maize primary roots in the seedling stage were analyzed by PEG treatment, and the results showed that the numbers of DEGs were increased as the treatment continued and that genes with GO categories “oxidoreductase activity” and “heme binding” regulated the deficit response [37]. Drought transcriptomes of maize ovary and leaf tissues were studied using RNA-Seq after 3 days withholding water, and the result indicated a positive response of ABA-related processes in the ovary leading to embryo abortion [32]. Compared with these studies with short-term drought (a few hours or days of drought treatment), the drought treatments in this study lasted 27 and 46 days. The most enriched GO categories of the tolerant line specific drought responsive genes and genotypic differentially expressed genes between tolerant and sensitive lines were stress or stimulus responsive, indicating that signal transduction pathways or drought responsive genes not only responded during the very early period of drought exposure, but also played important and sustained roles of adaption for longtime drought in the field.

For example, CBLs (Calcineurin B-like proteins) are the major types of Ca^2+^-sensor proteins to perceptual and integrated Ca^2+^ signals [77]. CBLs, along with CIPK (CBL-interacting protein kinase), regulate the responses to abiotic stresses at the transcriptional level and offer candidates for stress tolerance improvement in plants. The overexpression of *OsCIPK03*, *OsCIPK12* and *OsCIPK15* could improve the tolerance to drought, cold and salt stress [78]. The suppression of *OsCIPK23* by interference showed a hypersensitive response to drought stress in rice, and the overexpression could induce a number of drought-related genes, such as *DREB2A*, *Rab18* and *rd29A* [79]. The interaction of *AtCIPK1* with *AtCBL1* and *AtCBL9* regulated stress responses in an ABA-dependent and ABA-independent pathway [80]. In maize, *ZmCIPK16* was highly induced by drought, dehydration, salt and heat stresses, which could interact with *ZmCBL3*, *ZmCBL4*, *ZmCBL5* and *ZmCBL8*, yielded enhanced salt tolerance in overexpressed *Arabidopsis* [81]. In our study, three CIPK genes were highly expressed in the sensitive line under both moderate and severe drought. For instance, GRMZM2G113967, whose orthologous gene *AtCIPK14* has been reported to regulate the responses to salt and ABA treatments [82].

In conclusion, a number of drought responsive genes and genotypic differentially expressed genes between genotypes were analyzed by the RNA-Seq approach using two maize inbred lines with contrasting drought tolerance. Different regulation patterns and functional enrichment of these genes between the tolerant and sensitive lines were obtained. The overrepresented transcription factors in the tolerant line underlined their important roles in drought tolerance and adaption. Our results help to elucidate the drought responsive molecular mechanisms and provide candidate genes for further study to improve drought tolerance in maize.

## Supporting information

S1 Fig Correlation analysis of the transcriptomes of each replicate. The correlation of each replicate transcriptome was analyzed by log_10_(FPKM + 1). The correlations of H082183 under moderate drought (**A**), moderate drought control (**B**), severe drought (**C**), severe drought control (**D**) and Lv28 under moderate drought (**E**), moderate drought control (**F**), severe drought (**G**), severe drought control (**H**) were plotted.
(TIF)

S2 Fig Overlap of stress-related genotypic differentially expressed genes under well-water and drought stress. MD and SD indicate moderate drought and severe drought, respectively. MC and SC indicate well-watered controls of moderate drought and severe drought, respectively.
(TIF)

S3 Fig qRT-PCR validation of differentially expressed genes in H082183 and Lv28 under drought stress. The expression of eight randomly selected genes under moderate drought (MD), severe drought (SD) and their well-watered controls (MC, SC) were monitored by qRT-PCR. The fold change was calculated by MD/MC and SD/SC. The log_2_(fold change) values obtained by qRT-PCR and RNA-Seq were compared. The result showed a high similarity of gene expression trends (up- or down-regulated) between the gene expressions detected by the two methods.
(TIF)

S1 Table Statistical analysis of the RWCs between two water treatments of the drought tolerant and sensitive lines. (XLSX)

S2 Table Sequencing data output of each sample. (XLSX)

S3 Table Reference genome mapping results of each library. (XLSX)

S4 Table Expression of 462 common drought responsive genes of the two genotypes. (XLSX)

S5 Table List of the 19 genes that commonly responded to drought in H082183 and Lv28 under both moderate and severe drought. (XLSX)

S6 Table List of drought responsive genes that showed opposite regulation between the two genotypes. (XLSX)

S7 Table Common genotypic DEGs between the two lines under all water conditions. (XLSX)

S8 Table Stress-related genotypic DEGs. (XLSX)

S9 Table The 312 stress-related DEGs under both moderate drought and severe drought. (XLSX)

S10 Table Expression of the 20 enriched genotype-specific drought responsive transcription factors in H082183. (XLSX)

S11 Table Enrichment test of transcription factors in the H082183-specific responsive genes and genotypic DEGs under drought. (XLSX)

S12 Table Primers of the differentially expressed genes used for qRT-PCR. (XLSX)

## Abbreviations

H: Drought tolerant line H082183
L: Drought sensitive line Lv28
MD: Moderate drought
SD: Severe drought
MC: Well-watered control of moderate drought
SC: Well-watered control of severe drought
HMD: H082183 under moderate drought
LMD: Lv28 under moderate drought
HSD: H082183 under severe drought
LSD: Lv28 under severe drought
HMC: H082183 under well-watered control of moderate drought
LMC: Lv28 under well-watered control of moderate drought
HSC: H082183 under well-watered control of severe drought
LSC: Lv28 under well-watered control of severe drought
DEG: Differentially expressed gene
H+ genes: Genes up-regulated in H082183 comparing with Lv28
L+ genes: Genes up-regulated in Lv28 comparing with H082183
TF: Transcription factor
