# Supplementary figures and images for: Genome-wide identification of gene expression in contrasting maize inbred lines under field drought conditions reveals the significance of transcription factors in drought tolerance
Source: PLoS One. 2017 Jul 12;12(7):e0179477. doi: 10.1371/journal.pone.0179477 (PMC5507481; doi:10.1371/journal.pone.0179477)

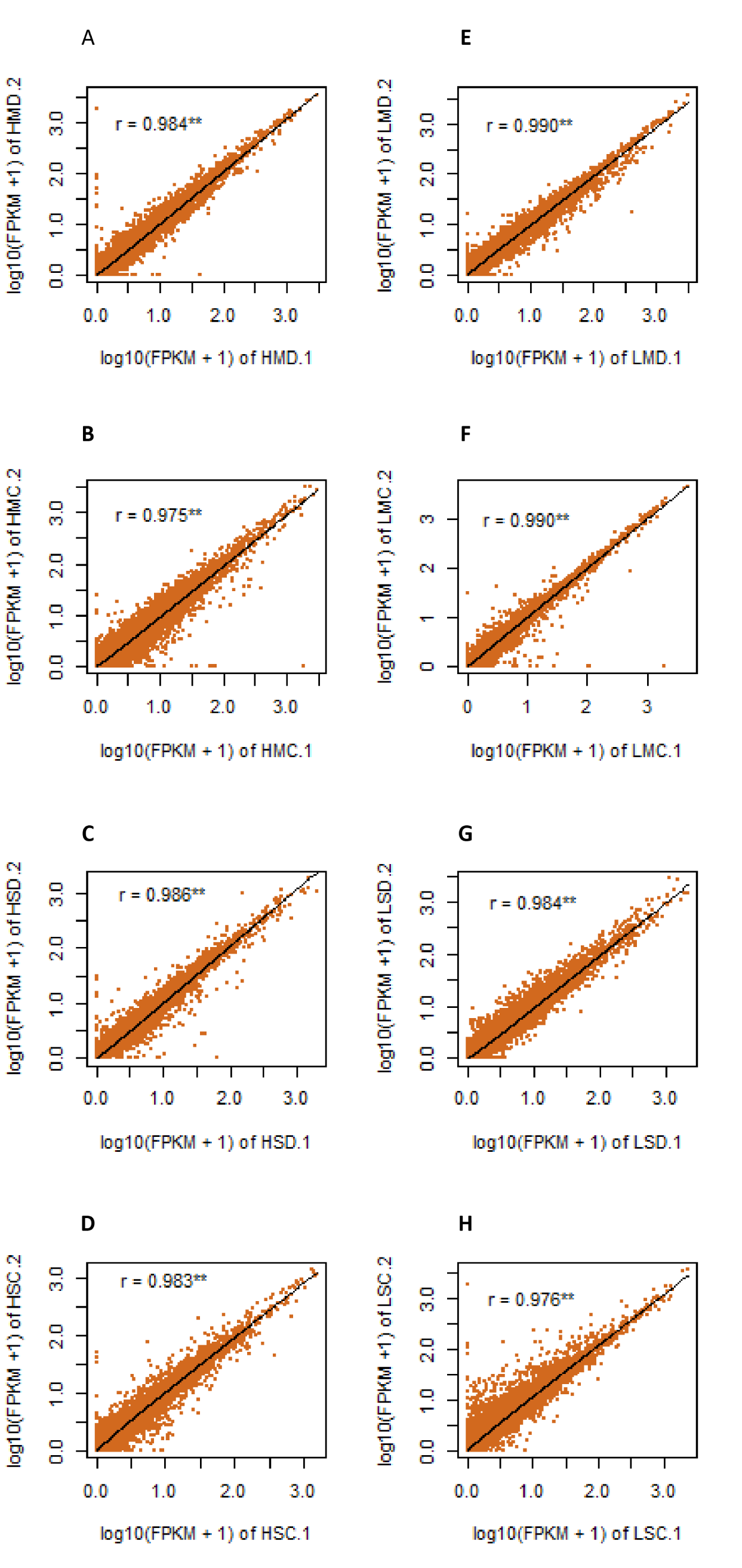

Supplement: S1 Fig — The correlation of each replicate transcriptome was analyzed by log10(FPKM + 1). The correlations of H082183 under moderate drought (A), moderate drought control (B), severe drought (C), severe drought control (D) and Lv28 under moderate drought (E), moderate drought control (F), severe drought (G), severe drought control (H) were plotted. (TIF) [file pone.0179477.s001.tif]

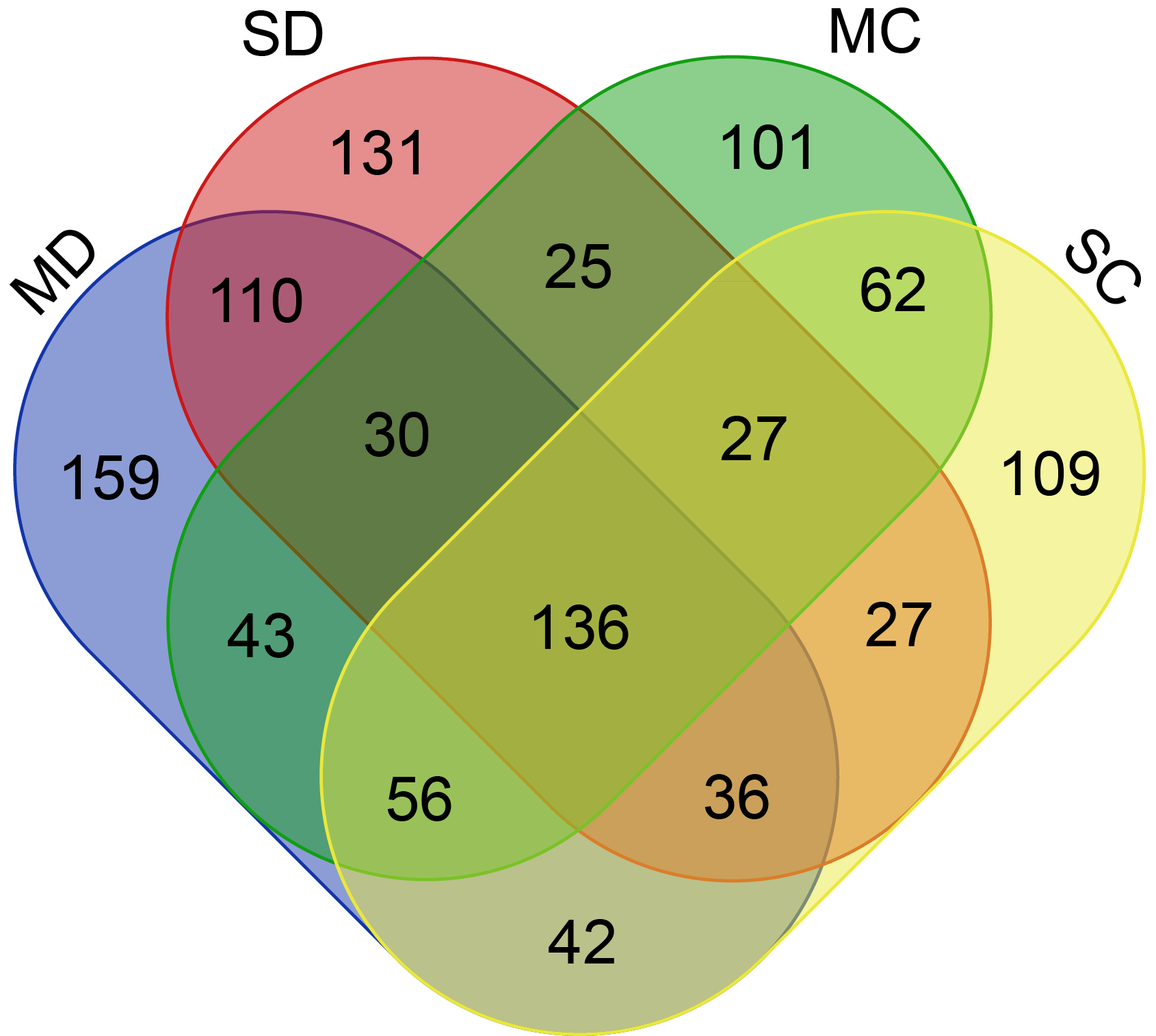

Supplement: S2 Fig — MD and SD indicate moderate drought and severe drought, respectively. MC and SC indicate well-watered controls of moderate drought and severe drought, respectively. (TIF) [file pone.0179477.s002.tif]

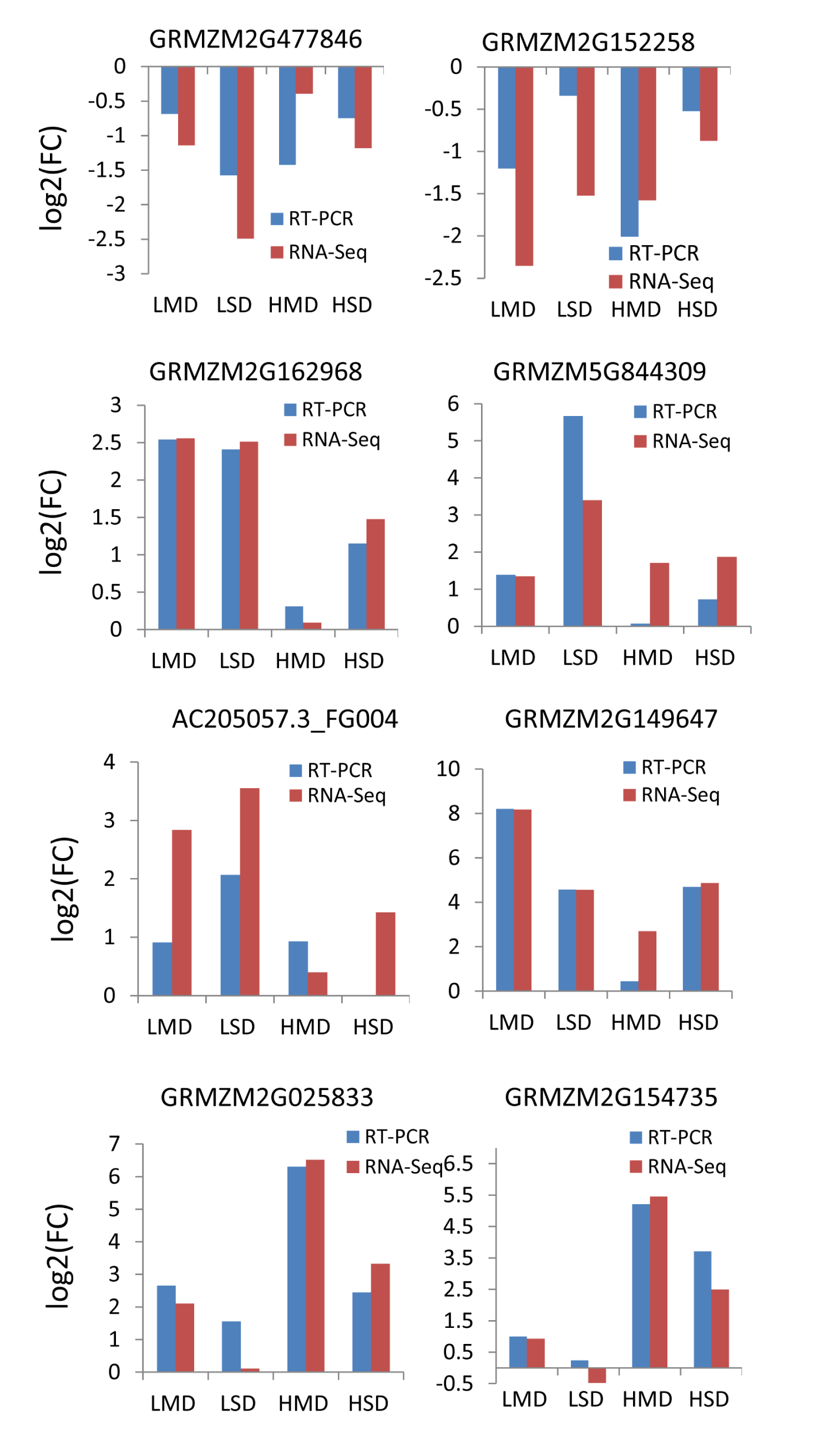

Supplement: S3 Fig — The expression of eight randomly selected genes under moderate drought (MD), severe drought (SD) and their well-watered controls (MC, SC) were monitored by qRT-PCR. The fold change was calculated by MD/MC and SD/SC. The log2(fold change) values obtained by qRT-PCR and RNA-Seq were compared. The result showed a high similarity of gene expression trends (up- or down-regulated) between the gene expressions detected by the two methods. (TIF) [file pone.0179477.s003.tif]
